# A co‐created multimethod evaluation of recovery education in Ireland

**DOI:** 10.1111/hex.13937

**Published:** 2024-03-28

**Authors:** Ann O'Brien, Louise Murphy, Amanda Hunt, David Dwyer, Andrew Hunter

**Affiliations:** ^1^ Business Information Systems, J. E. Cairnes School of Business & Economics University of Galway Galway Ireland; ^2^ Department of Nursing and Midwifery, Health Research Institute University of Limerick Limerick Ireland; ^3^ East Galway Recovery College (REGARI) Roscommon Ireland; ^4^ Recovery College Southeast Kilkenny Ireland; ^5^ School of Nursing and Midwifery University of Galway Galway Ireland

**Keywords:** co‐production, mental health, recovery education

## Abstract

**Background:**

This paper aims to explore the impact of recovery education on recovery knowledge, attitudes and the quality of life of students undertaking recovery education, contributing to the evidence base in relation to the impact of recovery education. It also explores the experiences of all stakeholders involved in the co‐facilitation, delivery and participation in recovery education.

**Setting and Participants:**

This study evaluates the experiences of stakeholders involved in the co‐facilitation, delivery and participation in recovery education across four recovery colleges in Ireland. Participants included students undertaking recovery education, peer educators, education facilitators, recovery college coordinators and practitioner/service providers.

**Discussion:**

Findings from the quantitative survey when compared with extant literature suggest that students had a good understanding of recovery education. The social aspect of empowerment for growth and wellbeing was identified through themes relating to co‐production and facilitating student learning. Support for equitable access to recovery education, including co‐production for both the public and staff, was identified as a challenge for the future.

**Conclusion:**

The findings from both the qualitative and quantitative components of the study show the positive impact of recovery education on stakeholders while acknowledging the need for ongoing support for people working in recovery education and the development of services. In particular, there was a high level of recovery knowledge found in students undertaking recovery education.

**Patient or Public Contribution:**

This study utilised a co‐created study design. From inception a steering group comprising stakeholders (peer educators, recovery education facilitators including past recovery college students and nonpeer staff involved in the co‐production of recovery education) directed the conduct of the evaluation. This steering group participated in an iterative process of information sharing, suggestions for evaluation process and language.

## 1 INTRODUCTION

Mental Health Ireland (MHI) is Ireland's longest established mental health charity, focusing on supporting people with lived experience of mental health. Together they collaborated with the Nursing and Midwifery Planning and Development Unit, the National Office of Mental Health Engagement and Recovery and the University of Galway to conduct this mixed‐methods evaluation study of the impact of recovery education in Ireland. Over the past number of years, the Health Service Executive (HSE), the body that runs all public health services in Ireland, has worked collaboratively with MHI. Working with people with lived experience of mental health challenges, along with family members, carers, supporters, service providers and subject matter experts. This collaboration has resulted in the co‐production and implementation of recovery education services and colleges across the Republic of Ireland.

The National Framework for Recovery in Mental Health (2018–2020),^1^ Sharing the Vision: A Mental Health Policy for Everyone (2020)^2^ and the Mental Health Ireland Strategy, Mental Health for All—Hope, Strength and Action (2022–2024),^3^ reinforces the importance of recovery, recovery education, co‐production and recovery‐oriented services that support people to recognise and take responsibility for their own recovery, wellbeing and recovery journey. These key documents have influenced the development of recovery education and guided this study approach. Recovery education is co‐delivered by peer and nonpeer facilitators in recovery colleges or recovery education services. Recovery colleges and recovery education services are based on the principles of adult education rather than clinical or therapeutic models and are based on transformative learning, not training. Some of the defining features of recovery education and recovery colleges are that they are collaborative, strength based, person centred, inclusive and community focused. Individuals who attend recovery colleges are known as students. A student is anyone with an interest in mental health regardless of their experience of mental health.

Since their inception in Ireland in 2013, funding has been secured in community health areas to develop Recovery Colleges and Recovery Education Services around Ireland. The ethos of these recovery education services is best described as follows:

*Recovery education takes a strength and adult education‐based approach, which offers the choice to engage in learning opportunities. It is underpinned by the values of self‐direction, personal experience, ownership, diversity and hopefulness*, National Advanced Recovery Ireland Recovery Education Working Group.^4^, ^5^ See Appendix 1 for a list of terms and abbreviations.

## 2 BACKGROUND TO THE STUDY

As recovery education services began to become part of mental health policy internationally the Advancing Recovery in Ireland (ARI) project aimed to improve stakeholders (students, peer educators—people with a lived and/or family experience, education facilitators—paid employees who work in collaboration with peer educators, recovery college coordinators and practitioner/service providers) knowledge of and attitudes towards recovery. The ARI project found that more recovery training was needed and introduced the concept of peer support workers.^6^ Currently, a body of research exists on the effectiveness of recovery education short courses,^7^, ^8^ and family recovery online courses.^9^ However, there is still a limited understanding of the effectiveness and impact of recovery colleges in Ireland.^10^ The mixed‐methods approach used in this study reflects the call for further qualitative evidence on the impact of recovery colleges.^11^

The study took place within the timeframe of the coronavirus disease 2019 (COVID‐19) pandemic against the backdrop of the challenges of social isolation where individuals sought positivity in daily life and learning new skills, which included the use of technology for social interaction.^12^, ^13^ At this time, recovery education has been shown to have a positive impact on student mental health and wellbeing.^9^ These positive impacts include the development of a range of benefits including positive social and personal wellbeing impacts of community support and peer involvement and highlight the value of co‐production as an important principle of personal mental health recovery.^14^ Table 1 illustrates where, across the life of the study, key co‐production steps were undertaken. The time spent at the planning and preparation of the study was particularly useful, as this encouraged the openness and transparency of the remainder of the research process.^15^

**Table 1:** Co‐production stages.

Dates	Stage	Action	Input
February 2021—August 2021	Planning	Designing the research, creating the question, designing the strategy, choosing instruments/framework used—reviewing documentation	Steering group assembled with peer educators (former RC students), Mental Health, Nursing and National Health Agencies and researchers. An iterative process of information sharing to find a common language. Core group was a smaller operational group of researchers and peer educators.
September 2021–February 2022	Data collection	Recruiting participants and explaining the research	Peer educators lead explaining the purpose of the research to students. Together with researchers hold online sessions to answer questions about the research.
		Undertaking data collection	
November 2021	Data analysis	Discussing the survey findings	Stakeholders gather to explore the findings from the survey
March 2022		Discussing themes in qualitative data	Using the Toney et al. (2018) framework proposed by the peer educator implications of the findings discussed
May 2022–September 2022	Output	Reviewing report—drawing inferences from the findings	Ongoing from core and steering groups
November 2021–February 2023		Creating outputs: poster, plain language summary, report, papers	Ongoing from core and steering groups

See also Appendix 4 for membership of the groups recovery education enables individuals and groups to manage their mental health, build a life of their choosing, accomplish recovery and/or support a loved one on their recovery journey. The principle of recovery has underpinned Irish mental health policy since the publication of A Vision for Change in 2006 ^16^ and has been further re‐emphasised in the more recent policy Sharing the Vision: A Mental Health Policy for Everyone 2020 ^2^ which provides a ten‐year road map for the direction of mental health services in Ireland.

## 3 THEORETICAL AND METHODOLOGICAL APPROACH

This research aimed to evaluate the impact of recovery education for all stakeholders (peer educators, recovery education facilitators and nonpeer staff involved in the co‐production of recovery education along with students attending recovery education classes/modules). A pre–post, mixed‐methods design was planned across a 6‐month ‘snapshot’ period of recovery education across Community Healthcare Organisation 2 (CHO2) and CHO5. CHOs deliver services outside the acute hospital system in the Republic of Ireland and include primary care, social care, mental health, and health and wellbeing services. In practice, implementation of this plan required modification on the basis that the majority of the initial survey participants had utilised recovery education on previous occasions. The focus groups and surveys were underpinned by the co‐produced change model for recovery education^17^ consisting of empowering environment, enabling different relationships/power and personal growth. Co‐production is at the heart of recovery education and there are increasing calls to undertake research mindful of this basis.^18^ Therefore, a co‐production approach underpins this evaluation with a view to adding to the evidence base, which utilises stakeholder's experience.^19^ Sustained recovery involves a wide range of social, educational and practical supports.

Co‐production is where key stakeholders work together to deliver these supports.^14^ Through co‐production, the process of designing, delivering and participating in recovery education is enhanced by the inclusion of the experience of people with lived experience, family members and service providers. Each participant and group bring their own expertise and skills to add to the overall expertise and knowledge available through recovery education. Recovery education can assist people in reclaiming personal power in and through learning to become an expert in their own wellness and taking back control over their own life. By participating in recovery education students can identify what supports and resources are needed to assist recovery and know how to access/avail of these supports/resources. Recovery education is recognised as a key driver for developing recovery‐oriented mental health services, which is highlighted in the HSE National Framework for Recovery in Mental Health 2018–2020.^1^ At the heart of this framework is the need to monitor the implementation of actions including co‐production and recovery education.^10^ A mixed‐methods approach was indicated by the co‐production stakeholders to maximise participation and ensure understanding of both the impact of recovery education and the mechanism.

## 4 STUDY AIMS

The overall research aim, as outlined by the project steering group, was to explore the impact of recovery education on the knowledge, attitudes/perceptions of individual recovery and overall experience of students after undertaking recovery education. In addition, this study sought to explore the experiences of all stakeholders involved in the co‐facilitation, delivery and participation in recovery education.

The main objectives of the study were to:

1. Identify the impact of recovery education on the recovery knowledge and attitudes/perceptions of people undertaking recovery education.
2. Evaluate the experience of all stakeholders involved in delivery, co‐facilitation and participation in recovery education.
3. Develop clear recommendations to support future research and developments for recovery education delivery in Ireland.

## 5 STUDY DESIGN

**Figure 1:**
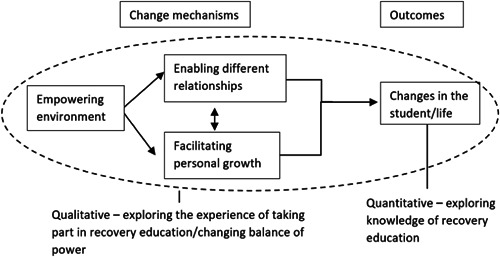
Overview of the study design influenced by the Toney et al. (2018) change model.

This study utilised a co‐created mixed‐methods study design. It was granted ethical approval from the relevant HSE Ethics Committees (Ref: C.A. 2691, JH/MV 20210906, 10/11/21). All participants were informed of their right to withdraw from the study at any stage without prejudice or penalty or infringement on their rights. The interviews were undertaken by trained mental health professionals well versed in local policies if a participant become distressed during the interviews as there is a potential for distress. The interviewers were academic researchers, not affiliated with the recovery colleges. Data collection sites included four recovery colleges in the South and West of Ireland. Quantitative and qualitative data was collected to provide a representative overview of recovery education in Ireland from the perspective of all stakeholders. Participants included students undertaking recovery education and all stakeholders involved in the delivery of recovery education in two representative Community Health Organisation sites in Ireland through recovery colleges (Figure 1).

Focus group analysis utilised a qualitative study design using a reflexive thematic analysis as described by Braun and Clarke's framework.^20^ The qualitative data from the focus groups was transcribed verbatim and qualitative data was managed using NVivo 12 software. The focus group discussion topic guide development was underpinned by the four classifications from the Recovery Colleges Characterisation and Testing (RECOLLECT) study^17^ (see Appendix 2). The application of the four classifications allowed grouping of questions in the focus groups. Ten focus groups were conducted, which consisted of: four student focus groups (*n* = 12), one peer educator group (*n* = 4), three groups of education facilitators/former students (*n* = 15), one group of recovery coordinators (*n* = 3) and one practitioner/service provider group (*n* = 2). The focus groups varied in size according to the availability of participants within each of the groups. Analysis indicates that the size disparity was offset by participants in smaller group's ability to provide more information. Focus group participants were recruited by the steering group representatives in the participating recovery colleges. Focus groups varied between 50 and 90 min in length and the decision to stop undertaking focus groups was made in conjunction with the peer educators during the ongoing analysis and identification of themes (see Table 1).

The analysis then focused on the data from this initial survey of 94 participants; in this initial survey, two scales were used to explore recovery knowledge and attitudes with students undertaking recovery education. The scales used were the popular Recovery Assessment Scale (RAS)^21^ and an amended (updated to reflect the language of recovery) version of the Recovery Knowledge Inventory (RKI).^22^ The online surveys were circulated by email and WhatsApp through the colleges and were mentioned in class. A nested parallel synthesis (see Appendix 3)^23^ consisting of data integration during interpretation phase after quantitative and qualitative data analyses was used (see Table 2).

**Table 2:** Data analysis.

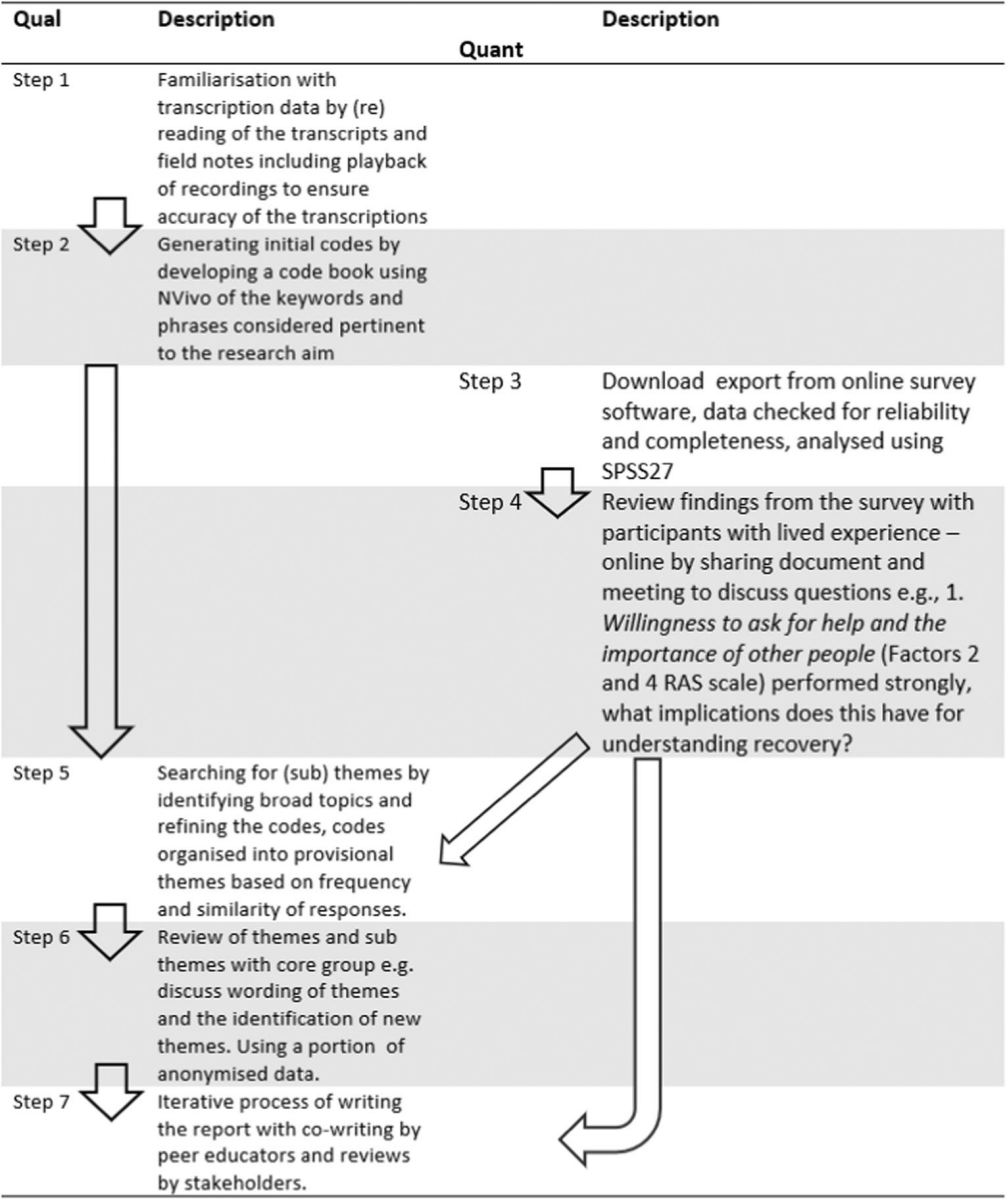

## 6 FINDINGS

### 6.1 Focus group findings

This study addresses the lack of evidence of *recovery experience* in Ireland by gathering data from stakeholders who participated in online focus groups describing their experience of taking part in recovery education. The evaluation has been undertaken, cognisant of the framework of the co‐produced change model of recovery education outlined by Toney et al.^17^ and the themes outlined below build upon that framework.

Four key themes and their supporting subthemes emerged from the analysis of all the focus groups (see Table 3): Qualitative thematic framework. The themes: *Empowering environment*, *Relationship* enhancement and engagement: Co‐production: *Enabling different relationships* and *Facilitating student knowledge, growth and wellbeing* are explored, with particular attention to the impact of recovery education on the balance of power as described by the participants. The final theme *Future structures* considers issues of ongoing capacity and structures relating to the provision of recovery education.

**Table 3:** Qualitative thematic framework.

Themes	Subthemes
Empowering environment	Elements of the social empowering environment Perception of a safe environment Finding voice
Co‐production: Enabling different relationships	Relationships with mental health services Peer support Education
Facilitating student knowledge, growth and wellbeing	Learning and applying knowledge Perspective transformation Volunteering
Future structures	Recovery education online in COVID times Future proofing recovery education Structural inequalities and national coordination

The four main themes are presented below along with supporting quotations from participants to explain and illustrate participant's experience of recovery education.

#### 6.1.1 Empowering environment

This theme explicates elements of the social experience of participants in recovery education services as safe spaces where students get the opportunity to develop their own voice. The data shows how individuals who first experienced recovery education as students would become empowered to develop, in some cases becoming peer educators and facilitators. The data shows recovery colleges creating physical and social settings within which students can develop and learn at their own pace.

There is just no judgement and so there is just this level of trust and understanding. That I've not found anywhere else. And I've been through the mental health system for nigh on thirty years. (FG7 L student)

Students and facilitators described how recovery college routines and processes helped them to feel comfortable sharing their own lived experience without fear of judgement or stigma. The respectful environment and openness of facilitators paved the way to safe sharing:

And then I met, I got involved in the atmosphere, it was such a buzz, from what I was used to, all I was getting beforehand was medication and OPDs. And suddenly I was involved and I was part of a community, the recovery movement. And it kind of, it opened up a whole lot of opportunities for me. (FG5 M former student now facilitator)

There is such an emphasis put on individual rights and respect. And like you say and the facilitators just lead with their own openness. It does help set that. It helps you feel that little bit more comfortable that you know these people aren't judging you know. So and then once, it starts a domino effect, because once they share, everybody shares. (FG7 L student)

Being able to voice and to discuss personal details of their experience of mental health difficulties without fear of stigma was one of the most important benefits of the recovery college environment:

The fact that it's a lived experience is the best I've ever known really. That I mean there is still a bit of a stigma with psychiatric illness. And if you have lived through an experience you know people will understand you know exactly where you've been. And do you know what I mean that you are, you know, how you lived through it. And how you survived it. (FG7 P student)

#### 6.1.2 Co‐production: Enabling different relationships

The theme Co‐production: Enabling different relationships further illustrates how within the Empowering environment offered by recovery colleges, the application of co‐production positively impacts on relationships, support and education. The student quoted below is clear that the facilitators create relationships that are equitable and enabling:

The facilitators make it very, very clear since the beginning that we are all equal. That our experience and our opinions are as valid as someone else … We have the right to have our opinions and to share our opinions with them. And yes, they are the experts, but as they as they are the experts by their study. But in another way, we are the experts by experience. (FG10 Student)

As noted, participants of all types spoke of the vital importance of co‐production, crucially participants were very clear regarding the centrality of co‐production and the factors supporting it. The different and encouraging nature of co‐production and the relationship with peers is noted by this student:

I have been asked to co‐develop the next course that is coming up in December. It's a three‐week workshop. I'm hoping to, I just said I don't know much about it, but I can give you my input and they said yeah absolutely we'd love to hear your input. (FG4 T Student)

Similarly, this exchange between peer educators illustrates how they value and support co‐production:

D: Yeah and in those moments you say thank god for co‐production (laugh).

V: And team work.

A: Yeah.

D: I suppose as well in terms of the push and pull like, so with the recovery facilitators you'd have a role in kind of guiding them and not minding them because they can mind themselves of course but just kind of looking out for them. (FG3 peer educators D, V and A)

This student notes the importance of being asked, involved as an equal and having their experience valued alongside other stakeholders, such as service providers and facilitators. Analysis of the data also indicates that while there is a clear co‐production benefit to developing relationships with mental health services, the service providers themselves can and do benefit from working with recovery colleges. Students with lived experience of mental health challenges describe how interacting with healthcare professionals in the recovery college environment helps to break down barriers and build trust:

I've encountered people who haven't been very lucky with the professionals and psychiatrists and there's just so much distrust that I think can grow that recovery colleges make huge or are very beneficial for these people and can also build up a trust again in professionals because a lot of these courses will have a few lived experience leaders and a few professionals and you really start to lose a sense that these are different people. (FG4 L Student)

When considering the data around co‐production and working with mental health services, the positive impact of developing these relationships, specifically for people with lived experience of less positive aspects of mental health service provision, was clear.

When discussing recovery education participants noted that the ‘tone’ being established in the recovery colleges allowed them to establish themselves as equal and participate:

So they set the tone. So because they're open, they're relaxed, they're happy to talk about their experiences and their dark places. You know that and I think that, like say helps to really set the tone for the whole kind of session. I mean I think that was I think the biggest thing that kind of surprised me. (FG7 Student)

Safe facilitation of co‐production stood out as the key component of this theme that allowed students to receive and in time participate in recovery education, finding and utilising their own confidence and voice.

#### 6.1.3 Facilitating student knowledge, growth and wellbeing

Students describe the initial impact of transformative learning in recovery education as a lightbulb moment, an entirely new perspective of hopefulness and change. Recovery education is designed in a similar way to adult education as it highlights the importance of the development of self‐agency and autonomy. This approach is supported by an empowering, stigma‐free environment and peer support that highlights the importance of lived experience:

It's a really nice place to grow from. (FG4 Y student)

Examples of participant's experience of transformative and empowering change were identified in the data:

20 years ago, I was told I'd be institutionalised for life. And now I've a whole life, I have friends, I have, and that keeps me well and I have a support system in the college that I can turn to any time and its totally confidential. (FG5 M former student now facilitator)

Importantly, recovery education students accept their own part in taking responsibility for their own learning and their own mental health.

… it differs from seeing a professional, you are kind of working on your own recovery rather than relying on someone else to help you through recovery. (FG4 K student)

Analysis of the data shows that recovery education provides practical tools and techniques for transformative learning and this process is enhanced through group dynamics involving peer support and group practices such as understanding the role of reflection:

… my life has improved in a way that I learnt techniques. And I learnt tools that I can use in my daily life. (FG10 M student)

Part of the recovery education process is learning practical skills and tools to support wellbeing. Students describe the hugely positive impact of the skills learned, and the life‐changing impact of these changes:

After a lot of different psychotic episodes my brain was kind of scattered and I kind of had to focus, I had to learn to focus and concentrate through the recovery college. And that's how its helped me in developing my social skills and my concentration and my memory. (FG5 M former student now facilitator)

A defining feature of recovery education described by students is developing self‐agency, the ability to take responsibility for their own learning. The data show how students are provided with the tools and techniques and the opportunity to make informed decisions:

You know they will guide you and they will offer you sort of signposts. But it's up to you to go and do the journey. To walk the walk. (FG7 L student)

… it brought enormous relief from the monotony of hearing people say you cannot do this and you cannot do that and you cannot do the other thing. (FG5 K former student now facilitator)

An acknowledged goal of transformative learning is *perspective change*. Students describe recovery education as a ‘game changer’, the mix of curated knowledge arising from co‐production that reflects lived experience and peer support opens a new vista of opportunity.

I never thought about being my own support. Because you're always looking outside for the supports. And when I, oh gosh and that's a big, big thing for me. (FG7 L student)

An instrumental element of recovery education is giving back, reflecting the example of their peer educators and many facilitators. Valuing the role as an expert volunteer emerged from the data as a theme that intersects across all themes. Where students discover the importance placed on lived experience and co‐production and organically identify with facilitators and peer educators with lived experience in the recovery education learning community:

There is something really nice in being able to pass on your knowledge you know and your little bits of wisdom that you've picked up from surviving mental health. Severe mental health problems you know. And again, I think it adds to your own sense of self‐worth. That so often you know I have felt just a waste of space, just useless, not capable of anything. That maybe there is some purpose that I've suffered the way I have suffered. That if that then can help somebody else to survive. (FG7 L student)

#### 6.1.4 Future structures

The theme Future structures considers issues around maintaining capacity and developing recovery education provision. Issues around supporting expertise in the recovery education services to undertake co‐production and strategic vision to support quality are addressed in this theme. Analysis of the data relating to Future structures indicates that recovery education is currently very successfully supporting people to change their lives for the better. Analysis though does note concerns relating to sustaining and supporting peer educators and facilitators.

Given that the data was collected between September 21 and March 23 responses to the COVID‐19 pandemic have influenced future structures in recovery education:

… a big highlight for recovery education in general has been the swift reaction to lockdown. We in (name of area) had no online presence one day and then within two to three days we were exclusively online because we had to be. (FG1 S former student now facilitator)

Despite the challenges, overall, the experience of moving online has been very positive, even described as a lifeline by some students. A student described how taking part in recovery education online overcame their travel difficulties:

… it can act like a steppingstone. There's a lot to be said for the comfort of your own home when you are starting something new. For a lot of people if they have any degree or anxiety about meeting new people or even the subjects that might be discussed in these workshops. Being able to start even at home online and during your own free time might encourage people to then start stepping out of the house and actually meeting up in person. I know it's done that for me. (FG4 Y student)

The reaction to COVID‐19 by recovery colleges and their use of online technology typifies much of the current practice in recovery colleges.

We had people that were from Norway, Africa, we got actually international you know. And that was a good highlight because you had Irish people, young people in their 20s traveling and they got caught in countries you know what I mean, and they were able to share. (FG1 B facilitator former student)

While the data indicate participants are positive about recovery education, there is a view that the established systems and structures cannot mature successfully until key structural challenges are addressed:

… the recovery college is you know it's worth its weight in gold but unfortunately it's not being given its weight in gold (FG1 P former student facilitator)

These challenges are encompassed by the interplay of sustainability and complexity, the findings represent an opportunity to address participant's concerns that recovery colleges do not have the capacity to fully utilise available expertise. These concerns were attributed to a lack of clear national policy implementation resulting in uncertainty of direction and inconsistency of provision:

Instead of having a sort of nationwide focal point for the development, the delivery of recovery education, where we're all doing our own. I mean when you think about it logically speaking, you know the pandemic and the virtual platforms, should have actually given us a complete opportunity to work together, you know with great ease. It has done the exact opposite and we're back to doing small groupings. (FG5 T facilitator former student)

This data is indicative of the participant's view that the established systems and structures cannot fully mature without structural challenges being recognised and addressed.

### 6.2 Survey findings

The survey aimed to find out who was taking part in recovery education in Ireland (*n* = 94) in these two sample areas, and discover the impact of recovery education on the recovery knowledge and attitudes/perceptions of people undertaking recovery education. Participants ranged in age from 18 to 86 years and over three‐quarters of respondents described themselves as female (*n* = 74). An overview of who participated is shown in Appendix 3.

#### 6.2.1 Who is participating in recovery education

One‐third of participants did not share their cultural background, and of the people who did, the overwhelming majority described themselves as Irish, White. Participants heard about recovery education from a variety of sources, with social media being popular in college 3 and college 2. The ‘Other health professional' option was the most popular in college 4; in this sample none of the participants heard about recovery education from their general practitioner (GP). Other ways that people heard about recovery education were from a college lecturer, local newspaper or poster, also mentioned were mental health forum, social prescribing service, volunteer website and website.

#### 6.2.2 Validity

Tests were conducted to ensure convergent and divergent validity of the data and these included findings for the RAS scale. The Cronbach's *⍺* was .93, for the RKI it was .75 and for all communalities it was over .5. The Kaiser–Meyer–Olkin measure of sampling adequacy was 0.8. Two well‐validated existing scales, the RAS^21^ and RKI,^22^ were used to discover a baseline for student's experiences of recovery education in Ireland. While causality cannot be claimed in this study, a considerable amount of prior literature refers to these scales; it was therefore possible to compare the results of this study with earlier findings.

Earlier research found that the mean of 28 studies for the RAS index using a 5‐part Likert scale ranged between 3.14 and 4.12.^24^ The mean score for RAS in survey 1 was 3.9 for all participants (*n* = 94), and despite the diverse range of experience of recovery education, this score is on the higher side in comparison to other studies (see Appendix 3).

The findings indicate that even participants who were early on their recovery journey (who had attended a few sessions of recovery education) with a recovery college have been positively impacted by recovery education. This is particularly true of the findings from the second scale RKI, here the findings compare very favourably than the mean scores of medical students who participated in recovery education^25^ (see Appendix 3).

## 7 DISCUSSION

A strength of this study was that all aspects of the study including the design, approach, analysis, interpretation of the findings and recommendations made were co‐created and co‐produced with recovery colleges/recovery education services, people with lived experience including family/carer/supporter/service provider. The study's findings are similar to those of other studies that identified that co‐production that includes students as well as peer educators is effective.^14^, ^26^

Evidence from the survey suggests that the recovery education provided is high quality and well received by students. Respondents displayed good knowledge of roles and responsibilities in recovery and roles of self‐definition and peers in recovery, which are factors of the RKI scale. This is reflected in the importance of RAS scale factor's goal and success orientation and no domination of symptoms. These survey findings were supported by the manner in which students in the focus groups repeatedly described the positive impact of recovery education, especially in the supportive environment where students felt a sense of belonging and could interact freely in the knowledge that they are understood and their opinion is valued equally.

Mirroring this identification of the importance of peers and the environment of recovery, representatives from the colleges with lived experience on review of the survey findings recognised the social aspect of empowerment for growth and wellbeing and the importance of connection with others as illustrated by the themes Co‐production: Enabling different relationships and Facilitating student knowledge, growth and wellbeing.

Evidence of co‐production was found at each recovery education college or service; all students in the focus groups commented favourably on the co‐produced modules and their understanding that they did have or felt that they would have opportunities to co‐produce.

Future implementation of recovery education should remain mindful of issues around equality of access and support. Concerns over equity for experts by experience in the co‐production of nursing education^27^ are mirrored to an extent by the consideration of how volunteers (public and staff) are remunerated for co‐production/participation.

Future research could also explore what are the barriers to accessing recovery education and how these can be overcome amongst organisations/stakeholders/practitioners. From the focus groups, evidence exists that learning is spread across stakeholder groups, but access to recovery education for service providers/practitioners is not equitable. There is a need to further embed and promote existing recovery education within formal structures of the mental health services through increased awareness and recognition of recovery education, for example, further recognition of existing accredited recovery education training modules with protected time given for continuing professional development.

Limitations of the study includes the small‐scale, capturing recovery colleges/recovery education services in two representative CHO areas in Ireland only. Across the nine CHO areas and the National Forensic Mental Health Service in Ireland, there is a locally responsive range of recovery education provisions. This should be taken into account when interpreting and contextualising the findings of the study. While this study does provide insight into the impact of recovery education on the knowledge, attitudes/perceptions of individual recovery and overall experience of students after undertaking recovery education, further larger scale research is required to strengthen the evidence‐base relative to best practice in recovery education and to identify the positive benefits of approaches such as co‐production in larger populations. Despite plans for a pre and post survey method, not enough participants described themselves as new to recovery education in the first survey, the size of the sample areas and the timing (during COVID) may explain this.

## 8 CONCLUSION

In conclusion, the data and recommendations show that barriers to recovery education relate to the need for greater communication of strategic direction, national support/organisational commitment and coordination to create and implement national policies on support and volunteering. At the time of the study, the sustainability of recovery education services was a concern of students and staff.

Uncertainty centred on the extent that the successes on the ground were reflected in future plans for recovery education across the country. Students recognise recovery education services as trusted information providers. The evaluation indicates a capacity to build upon this current provision and develop, support and promote recovery college stakeholders, for example, students, peer educators, education facilitators and recovery college coordinators.

The findings from the study highlight the positive impact of recovery education for all stakeholders. In particular, there was a high level of recovery knowledge found in students undertaking recovery education. This analysis represents an opportunity to better address these issues with recommendations arising from rigorously collected and analysed stakeholder data, which is closely aligned to the key principles identified in the National Framework for Recovery in Mental Health 2018–2020.^1^ It should be noted that while these recommendations are aligned to the principles, some are more applicable to the internal structures of the recovery colleges and the governance within, and others are more applicable to mental health services provision and engagement.

## AUTHOR CONTRIBUTIONS

**Ann O'Brien**: Study design; data collection; analysis and preparation of manuscript. **Louise Murphy**: Study design; data collection; analysis and preparation of manuscript. **Amanda Hunt**: Analysis; review and preparation of final manuscript. **David Dwyer**: Analysis; review and preparation of final manuscript. **Andrew Hunter**: Study design; data collection; analysis and preparation of manuscript.

## CONFLICT OF INTEREST STATEMENT

The authors declare no conflict of interest.

## ETHICS STATEMENT

Ethics approval was obtained from the Health Service Executive, Galway University Hospital Ethics Committee (Ref.: C.A. 2691), Health Service Executive, Mayo University Hospital Ethics Committee (Ref.: JH/MV 20210906), Health Service Executive South‐Eastern Area, University Hospital Waterford Research Ethics Committee (Ref.: 10/11/21). All participants provided written or recorded consent before focus group participation. Online questionnaire participants consented by clicking a ‘I consent to participate in this study’ link before being able to access the questionnaire.

## Data Availability

The data that support the findings of this study are available from the corresponding author upon reasonable request. The data are not publicly available due to privacy and ethical restrictions.

## APPENDIX 1 TERMS AND ABBREVIATIONS

ARI Advancing Recovery in Ireland

CHO Community Healthcare Organisation

GP General practitioner

HSE Health Service Executive

MHI Mental Health Ireland

NMPDU The Nursing and Midwifery Planning and Development Unit

RECOLLECT Recovery Colleges Characterisation and Testing

SPSS 27 Statistical Package for the Social Sciences 27

SRF Service Reform Fund

RAS Recovery Assessment Scale

RKI Recovery Knowledge Inventory

## APPENDIX 2 DISCUSSION TOPIC GUIDE

**Table:**

Discussion topic guide
1. Empowering environment (safety, respect and supporting choices) Please say your name and briefly outline what modules you have used when you first speak. Can you please tell us what personal impact (if any) recovery education has had on you
2. Enabling different relationships (power, peers and working together) What is your experience of recovery education face to face or online during the pandemic and now how do you feel about going back face to face?
3. Facilitating personal growth and shifting the balance of power (co‐produced learning, identifying and working with strengths) Do you feel there is co‐production in your college?
4. Outcomes, change in the student (self‐understanding and self‐confidence) and changes in the student's life (occupational, social and service use) Have you been made aware of other organisations for education or support you can use?

## APPENDIX 3 PARALLEL SYNTHESIS

**Table:**

Settings	Data collection	Who
Four recovery education colleges in Two Community Health Organisations (Areas)	Online surveys	Invitations by peer educators in colleges to students Administered by researcher
One area had three colleges and the other had one college (hub and spoke design)	Focus groups— online (recovery education was online due to COVID restrictions at the time)	Invitations by peer educators in colleges to students Focus groups of distinct groups—for example, four groups of students (12 participants); one group peer educators (four participants); three groups education facilitators (15 participants^a^); one co‐ordinators group (three participants); one group of practitioners/service providers (two participants)—researcher interviewers

a Most education facilitators also had lived experience.

**Table:**

	Who was taking part						Gender	
Recovery college	Lived experience	Family only	Health practitioner/service provider	Health student	Lived experience and family	Lived experience and service provider	Female	Male
College 1	8	0	1	0	1	0	9	1
							12.2%	5%
College 2	11	6	3	1	5	4	24	6
							32.4%	30%
College 3	10	4	0	1	6	0	14	7
							18.9%	35%
College 4	10	1	8	5	5	4	27	6
							36.5%	30%
Total	39	11	12	7	17	8	74	20
**%**	41.5	11.7	12.8	7.4	18.1	8.5	77	23

Table 4#: Mean scores Recovery Assessment Scale			
		Mean	
Recovery Assessment Scale (RAS)			
Average of mean from 28 studies for the RAS^24^		3.78	
Average current study (all participants)		3.9	
RAS factors			
No domination of symptoms		3.4	
Goal and success orientation		4	
Willingness to ask for help		4	
Reliance on others		4.1	
Personal confidence and hope		3.8	

Table mean scores Recovery Knowledge Inventory		
	Feeney et al.^25^ (medical students)	Current study (recovery college students)
Recovery Knowledge Inventory	Mean	Mean
Roles and responsibilities in recovery	3.18–3.66^a^	3.6
Nonlinearity of the recovery process	2.23–2.38^a^	2.7
Roles of self‐definition and peers in recovery	3.71–3.93^a^	4.4
Expectations of recovery	2.55–3.28^a^	3.7

a Baseline and after recovery placement.

## APPENDIX 4 GROUP MEMBERSHIP

**Table:**

Steering group
Deputy Chief Executive Officer National Mental Health Organisation
Directors Nursing and Midwifery Planning and Development Units
National Engagement and Recovery Lead
Mental Health Engagement and Recovery National Health Service
Peer educator
Lead peer educator
Researchers university

Core group
Peer educator
Lead peer educator
Researchers university
